# Intrasynovial autograft for reconstruction of chronic large rotator cuff tears in a rabbit model: biomechanical, computed tomography, and histological results

**DOI:** 10.1186/s13018-024-04691-2

**Published:** 2024-04-04

**Authors:** Lazaros Kostretzis, Iosafat Pinto, Konstantinos Katakalos, George Kazakos, Angeliki Cheva, Pericles Papadopoulos, Konstantinos Ditsios

**Affiliations:** 1 2nd Orthopaedic Department of Aristotle, University of Thessaloniki, General Hospital of Thessaloniki “G.Gennimatas”, Thessaloniki, Greece; 2 Laboratory for Strength of Materials and Structures, Civil Engineering, Department of Aristotle, University of Thessaloniki, Thessaloniki, Greece; 3 School of Veterinary Medicine of Aristotle, University of Thessaloniki, Thessaloniki, Greece; 4 Department of Pathology, Faculty of Medicine, Aristotle University of Thessaloniki, Thessaloniki, Greece

**Keywords:** Large rotator cuff tear, Subscapularis tendon, Rabbit, Chronic tear, Intrasynovial, Flexor digitorum profundus, Biomechanical, Computed tomography, Histological evaluation, Reconstruction

## Abstract

**Background:**

Rotator cuff (RC) tears are a common cause of shoulder dysfunction and pain, posing significant challenges for orthopedic surgeons. Grafts have been proposed as a solution to augment or bridge torn tendons, but optimal clinical outcomes are not always achieved due to poor graft integration, suboptimal mechanical properties, and immunological reactions. The aim of this study was to investigate the biomechanical, CT and histological results of RC reconstruction using an intrasynovial tendon autograft, in a chronic large tear subscapularis rabbit model.

**Methods:**

Twenty-six adult male Zealand white rabbits were used in this study. Large defects in the subscapularis tendons were produced bilaterally in 20 rabbits. After 6 weeks, secondary procedures were performed to the right shoulder of the rabbits, which were reconstructed with an intrasynovial interposition autograft (graft group). The left shoulder did not undergo any further treatment (defect group). The specimens were randomly divided into two equal time groups and underwent biomechanical testing, CT analysis, and histological evaluation at 6, and 12 weeks after reconstruction. In addition, 6 rabbits that were not operated, were used as a control group.

**Results:**

At 12 weeks post-repair, the graft group exhibited a significant increase in ultimate failure load compared to the defect group (*p* < 0.05). Furthermore, the 12-week graft group demonstrated comparable stiffness to that of the control group. CT analysis indicated no significant progression of intramuscular fat accumulation in both graft groups, in contrast to the 12-week defect group when compared to the control group. Finally, histological evaluation revealed a gradual integration of the graft with the host tissue at 12 weeks.

**Conclusion:**

Our study suggests that intrasynovial flexor tendon autografts hold promise as an effective interposition graft for the reconstruction of chronic large RC tears, as they improve the biomechanical and biological properties of the repaired tendon. Nonetheless, further investigations in preclinical large animal models are warranted to validate and extrapolate these findings to human studies.

## Background

Rotator cuff (RC) tears are a prevalent cause of shoulder dysfunction and pain, often leading to impaired range of motion and diminished quality of life [1] while also placing an enormous economic burden on society [2]. Despite advances in surgical techniques and rehabilitation protocols, the management of large or massive RC tears remains a significant challenge for orthopedic surgeons. Retears of the RC repair have been documented to range from 16.7 to 57% for large or massive tears [3–5]. Different types of autografts, allografts, and ultimately synthetic and tissue engineered scaffolds have been described and introduced for the reinforcement of RC repairs with various outcomes [6].

When inadequate or frail native tissue exists, grafts can be used to augment or bridge the torn tendon. However, surgical reconstruction of tendons using grafts does not always result in optimal clinical outcomes. The main reasons for this include (1) poor graft integration; (2) suboptimal mechanical properties; and (3) inflammatory or immunological reactions against the graft [7]. Traditionally, autografts are considered the preferred choice [8], despite the potential cause of donor site morbidity. Autografts have certain advantages over allografts, including favorable biomechanical properties, lack of immunogenicity, fast incorporation, and low infection rates [8]. Clinical and laboratory studies have evaluated different types of autografts for the reconstruction of RC tears [9]. The use of intrasynovial autografts has been extensively studied in the challenging setting of hand flexor tendon (intrasynovial) injuries [10]. Clinical and laboratory studies have shown that for intrasynovial recipient sites, extrasynovial autografts have less favorable biologic effects because (1) a greater rate of matrix turnover is required to match the intrasynovial recipient environment [11]; (2) they usually undergo early cellular necrosis; and (3) repopulation by cells from the surrounding tendon often leads to peritendinous adhesions [12]. The rotator cuff tendons and their intrasynovial location biomechanically and biologically mimic the hand flexor tendons inside their synovial sheath [13].

Based on structural and metabolic differences that have already been observed with intrasynovial autografts [14, 15], we decided to study their healing potential when transferred to the shoulder joint to reconstruct RC tears. The rabbit animal model has been commonly applied to evaluate muscular changes associated with RC tears, including muscle fatty infiltration, and RC healing with or without graft reconstruction [16]. More recent data showed that the subscapularis (SSC) in rabbit study models better resembles the human anatomy of the RC and subacromial arch [17].

The objectives of this study were (1) to evaluate the biomechanical performance of the intrasynovial autograft for the interposition reconstruction of a chronic large RC defect, (2) to study the distribution and progression of fat accumulation along the muscle with computed tomography (CT) quantification, and (3) to assess the histological changes of the bone‒tendon enthesis after 6 and 12 weeks. We hypothesized that the intrasynovial autograft would enhance the biomechanical properties, hinder fat accumulation along the muscle and promote tendon-to-bone healing.

## Methods

This is a controlled laboratory study involving animals and was conducted following the ARRIVE guidelines (Animal Research: Reporting of In Vivo Experiments) [18]. All procedures and protocols were approved by the Institutional Animal Committee and the local Bioethics Committee.

### Animal study design

**Fig. 1:**
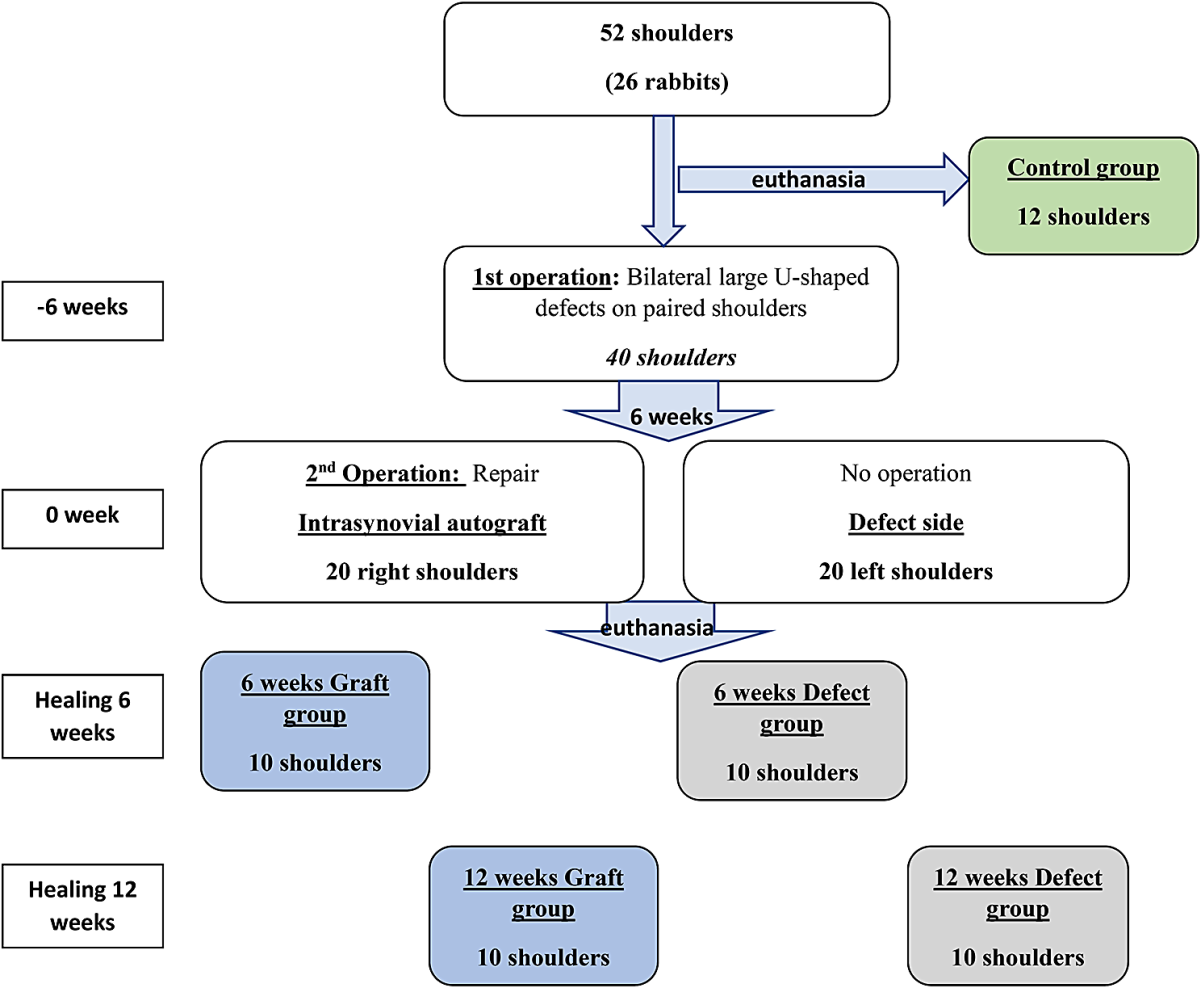
Diagram of the experimental process. For each group, 8 shoulders underwent CT analysis and sequential biomechanical testing, while 2 shoulders underwent histological evaluation. For the control group, 8 shoulders underwent CT analysis and sequential biomechanical testing, and 4 shoulders underwent histological evaluation

Twenty-six adult male New Zealand white rabbits (age, 12 weeks; weight, 2.9 ± 0.3 kg) were used in this study. Twenty rabbits underwent two operations, and 6 rabbits were not operated on, comprising a control group (Fig. 1).

In the first operation, 20 rabbits (in total 40 shoulders) were used to bilaterally create a large defect by surgically removing a part of the subscapularis (SSC) tendon. After 6 weeks, secondary procedures were performed only on the right shoulder of all rabbits, which were reconstructed with an intrasynovial interposition autograft (graft group). The left shoulder of all rabbits did not undergo any further treatment (defect group) to evaluate the spontaneous healing capacity. All rabbits were housed individually at 22 °C in their cages with free access to water and food and were allowed to freely move after surgery.

The specimens were euthanized, and bilateral shoulders were harvested randomly in two time periods: 10 rabbits at 6 weeks (6-week groups) and 10 rabbits at 12 weeks (12-week groups) after the second operation. Consequently, for each time group, 8 rabbits underwent CT analysis and sequential biomechanical testing, while 2 rabbits underwent histological evaluation. For the control group, 4 rabbits underwent CT analysis and sequential biomechanical testing, and 2 rabbits underwent histological evaluation.

### Anaesthesia, analgesia and antimicrobial prophylaxis

For each surgical procedure, rabbits were sedated a qualified veterinarian (GK) with dexmedetomidine 80 µg/kg im and tramadol 2 mg/kg im, anesthetized using propofol to effect, intubated and maintained with isoflurane. For analgesia and antimicrobial prophylaxis, meloxicam 1 mg/kg im SID and 150 mg cefamandole im SID were administered for 3 days, respectively.

### Chronic subscapularis large tear model

Two orthopaedic surgeons (LK and IP) performed all operations in sterile fashion under loop magnification and with the use of microsurgery instruments. A longitudinal anterolateral skin incision of approximately 3 cm was made over the ventral side of each shoulder, followed by dissection of the deltoid muscle in line with its fibers on its sternal side. The subscapularis insertion onto the lesser tubercle was then identified.

**Fig. 2:**
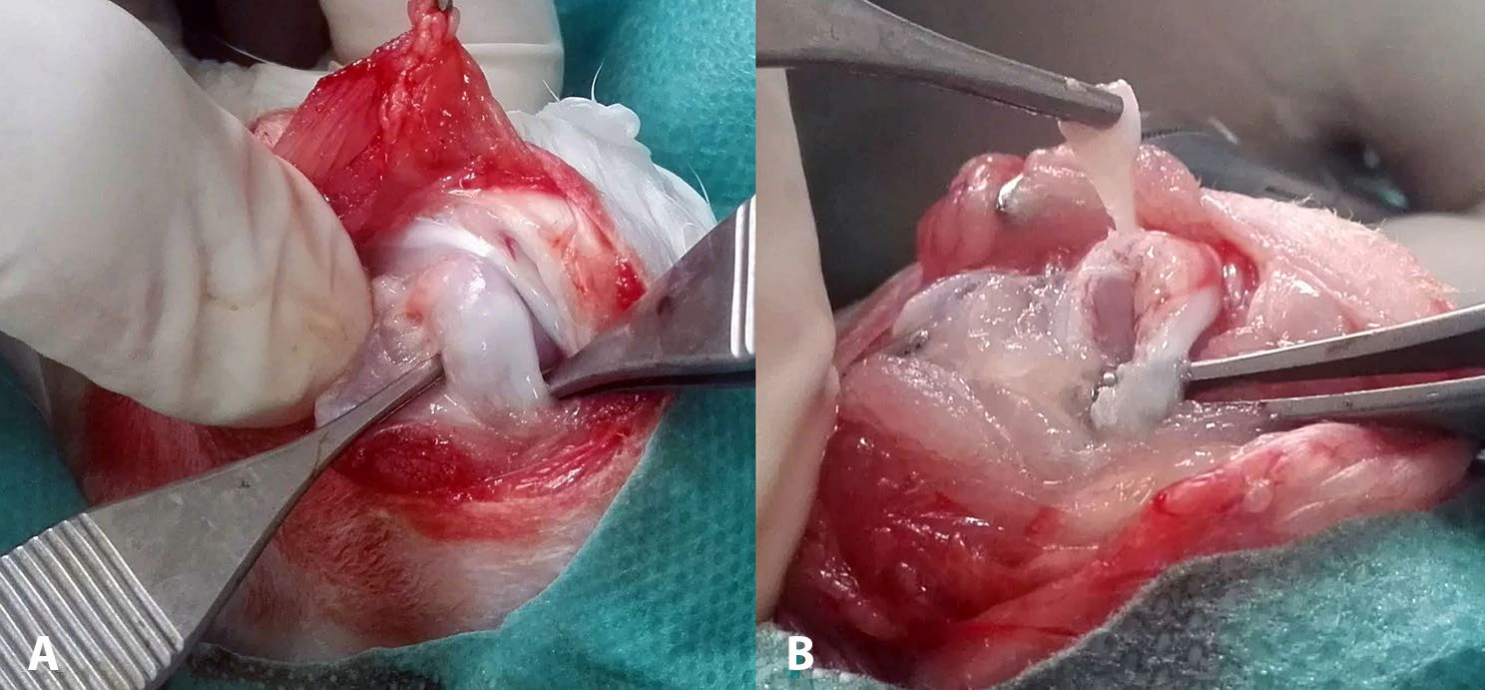
1st Operative procedure of (**a**) identifying the supraspinatus tendon and (**b**) creating a U-shaped defect

The subscapularis tendon of every shoulder was measured in thickness and width with a caliper, and then a full-thickness U-shaped defect from the insertion onto the lesser tuberosity was produced in the middle portion of the tendon by performing a sharp dissection with a No. 11 scalpel blade. The width of the U-shaped defect was approximately 50% of the width of the tendon and spanned in length from the footprint to the musculoskeletal junction. Finally, the wound was closed in layers (Fig. 2). Postoperatively, wounds were inspected daily for infection or dehiscence. A chronic large tear model was achieved after 6 weeks [19].

### Reconstruction of chronic large tears with an intrasynovial autograft (flexor digitorum profundus)

After 6 weeks, we approached again only the right subscapularis and lesser tuberosity. The footprint that corresponded to the U-shaped defect on the lesser tuberosity was prepared to a rough surface with bleeding with a small rongeur. Two bone tunnels (0.8 mm in diameter) were produced with a drill from the medial side of the footprint defect to the lateral side of the lesser tuberosity.

**Fig. 3:**
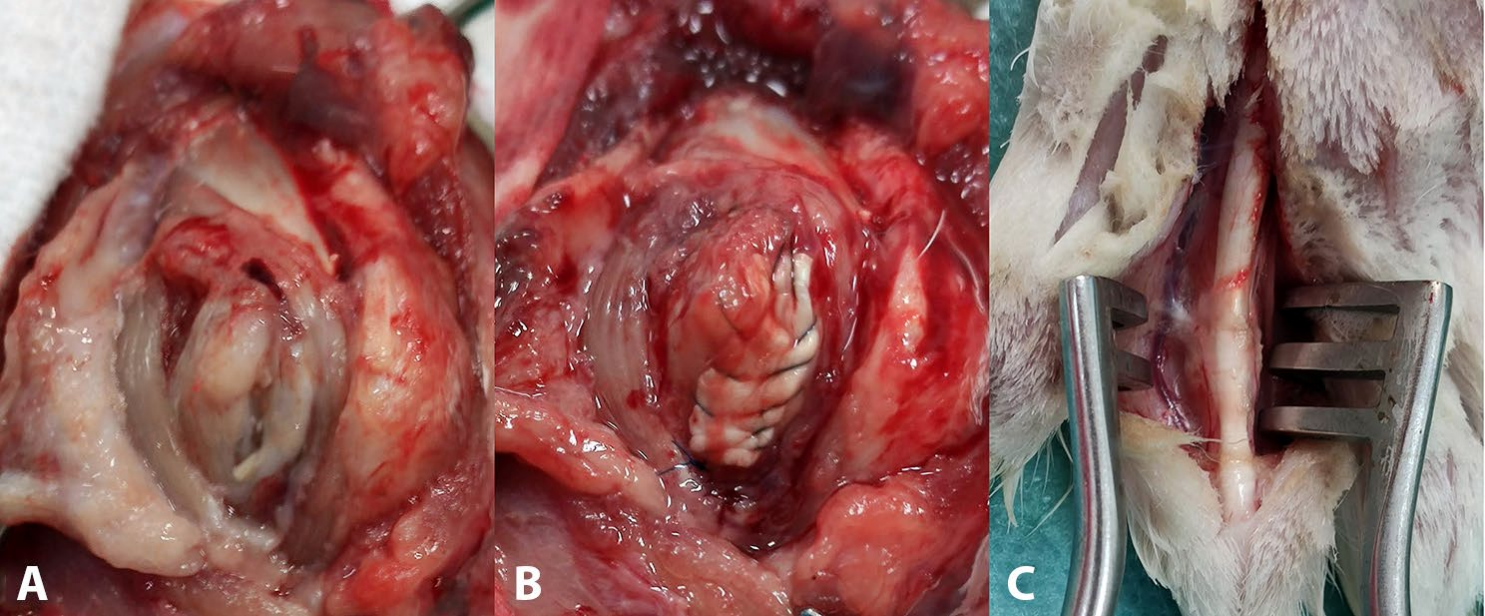
2nd Operative procedure of reconstructing (**a**) the chronic defect of the supraspinatus (**b**) with an intrasynovial autograft (flexor digitorum profundus). (**c**) The graft is harvested randomly from the 2nd or 3rd digit of the ipsilateral hind paw

The ipsilateral hind paw was also prepped and draped in a sterile fashion. A longitudinal incision was made randomly on the ventral side of the 2nd or 3rd digit, the flexor tendon sheath was opened, and a segment of approximately 8 mm of the tendon of the flexor digitorum profundus (FDP) distal to the A1 pulley was harvested. After that, the wound on the hind paw was closed. The graft was cut in size to fit the defect, and its distal end was attached to the lesser tuberosity. Specifically, a modified Kessler suture technique using a 5 − 0 (Prolene, Ethicon, USA) suture was placed to the distal end of the graft, the 2 limbs of the sutures were inserted in the 2 drill holes of the lesser tuberosity, and the suture limbs were tied together. With the same suture material that still had its needle attached, the graft was securely sutured circumferentially to the defect, and finally, the suture limbs were tied again, thus securely patching the defect (Fig. 3).

On the left shoulder, no operation was performed.

### Specimen collection

At predetermined time points (6 or 12 weeks after the second operation), rabbits were euthanized by an overdose of iv propofol. All specimens were obtained by harvesting the proximal humerus, the entire rotator cuff muscles and tendons, and the scapula. Bilateral shoulders were evaluated macroscopically for signs of infection, adhesions, and rotator cuff integrity. Specimens were excluded from the study if the animal died prematurely or if any signs of wound infection or dehiscence were present.

### Gross examination

Following euthanasia, all specimens were evaluated macroscopically to assess any absent, partial, or complete healing.

### Computer tomographic evaluation

All specimens that were used for biomechanical testing first underwent CT detection immediately after euthanasia in the radiology department in the facilities of the Companion Animal Clinic of the School of Veterinary Medicine. The images were obtained with Optima CT520 Series (GE Medical Systems) using CT Dose Index 3.14 mGy, 49 mA, 120 KV, and 1.25 mm collimation. The specimens were placed in the coil in the longitudinal position, and transverse imaging of the muscles of the rotator cuff was performed routinely. The goal was to quantify intramuscular fatty infiltration in the cross-sectional area (CSA) of the muscle of the subscapularis based upon the different Hounsfield unit (HU) ranges that define fat and skeletal muscle [20]; a lower value of HU (lower attenuation) indicated a greater amount of fat. Acquisition images were sent to a picture archiving and communication system (Radiant DICOM Viewer 2023.1) for analysis. These values were measured at three equal levels along the length of the muscle: the lateral quarter (proximal to the tendon), middle half, and medial quarter, which were defined by measuring the length of the muscle in the coronal reconstruction images. An experienced radiologist blinded to the identity of the specimens performed this analysis.

The attenuation signal was evaluated by calculating the mean HU value inside a 10-mm² circular region within the center of the muscle at each site [20].

The mean intramuscular fat signal was calculated as the mean value of the three sites.

### Biomechanical evaluation

**Fig. 4:**
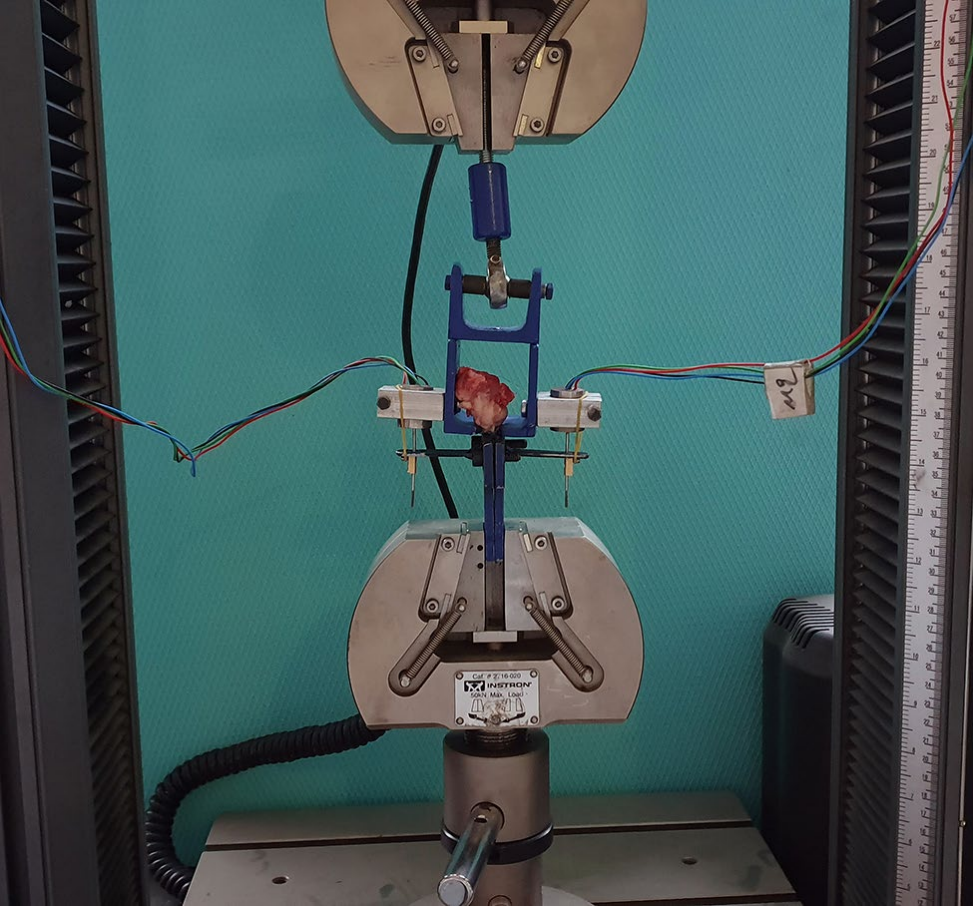
Biomechanical testing setup of the specimen

Following CT examination, the shoulders were examined biomechanically at the Laboratory of the Civil Engineering department. The shoulders were kept in a box with ice for transfer, and they were thawed at room temperature before testing. The humeral head and SSC tendon were prepared until no soft tissues remained attached to it. The tendon was grasped with 2 − 0 Ethibond (J & J Medical N.V., Belgium) sutures in a Krakow style 5 mm from its insertion to the lesser tuberosity to avoid delamination during pull. Next, the free end of the tendon and the suture were fixed in a holding clamp with grit sandpaper and commercial cyanoacrylate glue to prevent it from slipping during tensile testing. The humeral head was secured in a custom-made clamp. An Instron model 5969 (USA) testing frame was used to apply the demanded load to the specimens. Two linear variable differential transformers (LVDTs) (Micromeasurement, USA) were used separately to more precisely evaluate tendon elongation. All data were collected and recorded using the LSMS Data Acquisition System (DAS, Kyowa, Japan). The SSC tendon was fixed to this system along its anatomic direction (tendon to bone) to allow uniaxial tensile loading (Fig. 4). First, the tendon was pretensioned to 15 N for 30 s to precondition the construct for load-to-failure testing. After that, the tendon was loaded to failure (until it was pulled apart from the bone junction, ruptured at its midsubstance, or avulsed medially from the clamp). The rate of the imposed displacement was 1 mm/s. The ultimate failure load was defined as the peak force and was recorded automatically with a data acquisition system. Stiffness was computed by calculating the slope of the most linear portion of the curve from the load and displacement graph for each specimen. Data regarding the site of failure, ultimate failure load, and stiffness were further analyzed and compared among the different investigated groups and time periods.

### Histological examination

To evaluate tendon enthesis, the SSC tendon and muscle with the proximal humerus were fixed in 10% neutral buffered formalin for 24 h. After fixation, the tissues were decalcified in 6.5% nitric acid for 4 h and then embedded in paraffin blocks. Three-micrometer-thick serial sections were cut using a microtome and stained with hematoxylin-eosin to evaluate the general morphology and cellularity and Masson’s trichrome to evaluate the fibrous collagen structure.

All specimens were evaluated by an experienced histopathologist (AC) who was blinded with regard to specimen group. The cross-sections were examined under a stereoscopic microscope (Nikon Eclipse E600, Tokyo, Japan). Digital images were taken using a camera system for microscopy (Nikon model DS-Fi2-L2, Tokyo, Japan). The qualitative analysis of histologic results included organization of collagen tissue, vascularity at the tendon-bone interface, cellularity, inflammation, and collagen fiber continuity between tendon and bone.

### Power and statistical analysis

Ultimate load to failure in the biomechanical testing was chosen as the primary outcome of this study. The power analysis that was performed a priori (mean difference, 55 N; standard deviation of difference, 35 N; a- error, 0.05; b-error, 0.8) based on a previous study [21] indicated that a minimum sample size of 8 per group was required for the biomechanical analysis to detect a significant difference in ultimate failure load.

Statistical analyses were performed by using R Studio version 4.3.0 software package. Shapiro‒Wilk normality tests were used to determine the distribution of continuous variables. The statistical comparison of the biomechanical properties of each group was performed using the two-sample t test (pooled variance). The Mann‒Whitney U test was used to determine the relationship between biomechanical properties and site of failure. The Kruskal‒Wallis test using the Bonferroni correction, followed by the post hoc Mann‒Whitney U test, was used to compare the data from the CT analysis. Spearman’s rank correlation coefficient was used to evaluate any correlation between the CT attenuation signal and biomechanical data.

All data are presented as the mean ± SD, and the significance was set at *p* < 0.05.

## Results

### Gross examination

All animals demonstrated free full range of motion, without any signs of limping before euthanasia. No rabbits died before euthanasia, and there were no wound healing problems.

**Fig. 5:**
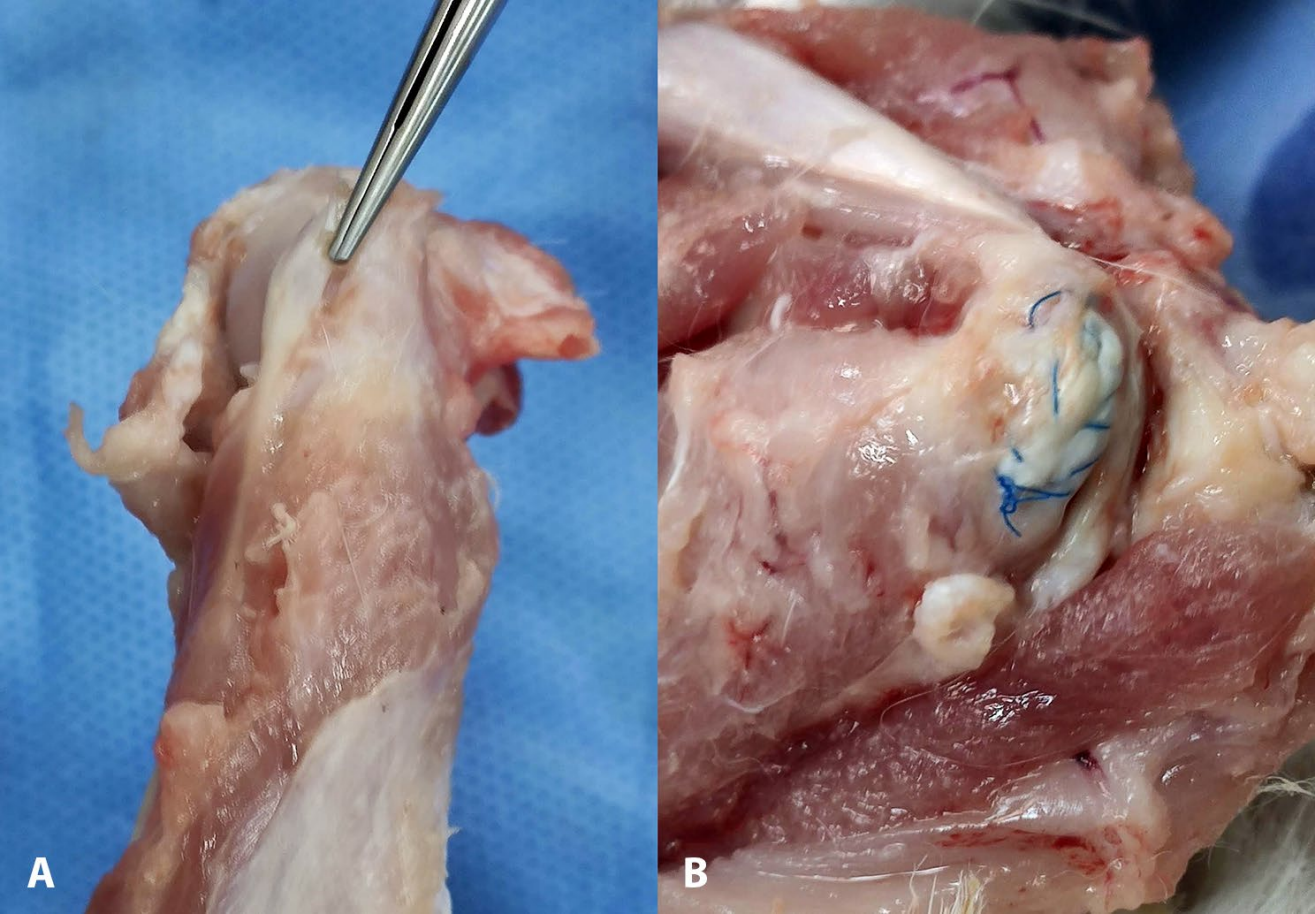
Gross morphological findings of the specimens 12-weeks after the 2nd operation (**a**) in the defect group and (**b**) in the graft group

At 6 weeks after reconstruction, the graft had developed sparse connective tissue with the host tendon, whereas on the defect side in all specimens, the defect was still visible. At 12 weeks after reconstruction, the graft had developed a dense connective tissue bridge with the host tendon, while on the defect side, very loose connective tissue had formed, and the defect was still clearly visible (Fig. 5).

### Biomechanical testing

#### Ultimate failure load and linear stiffness

**Fig. 6:**
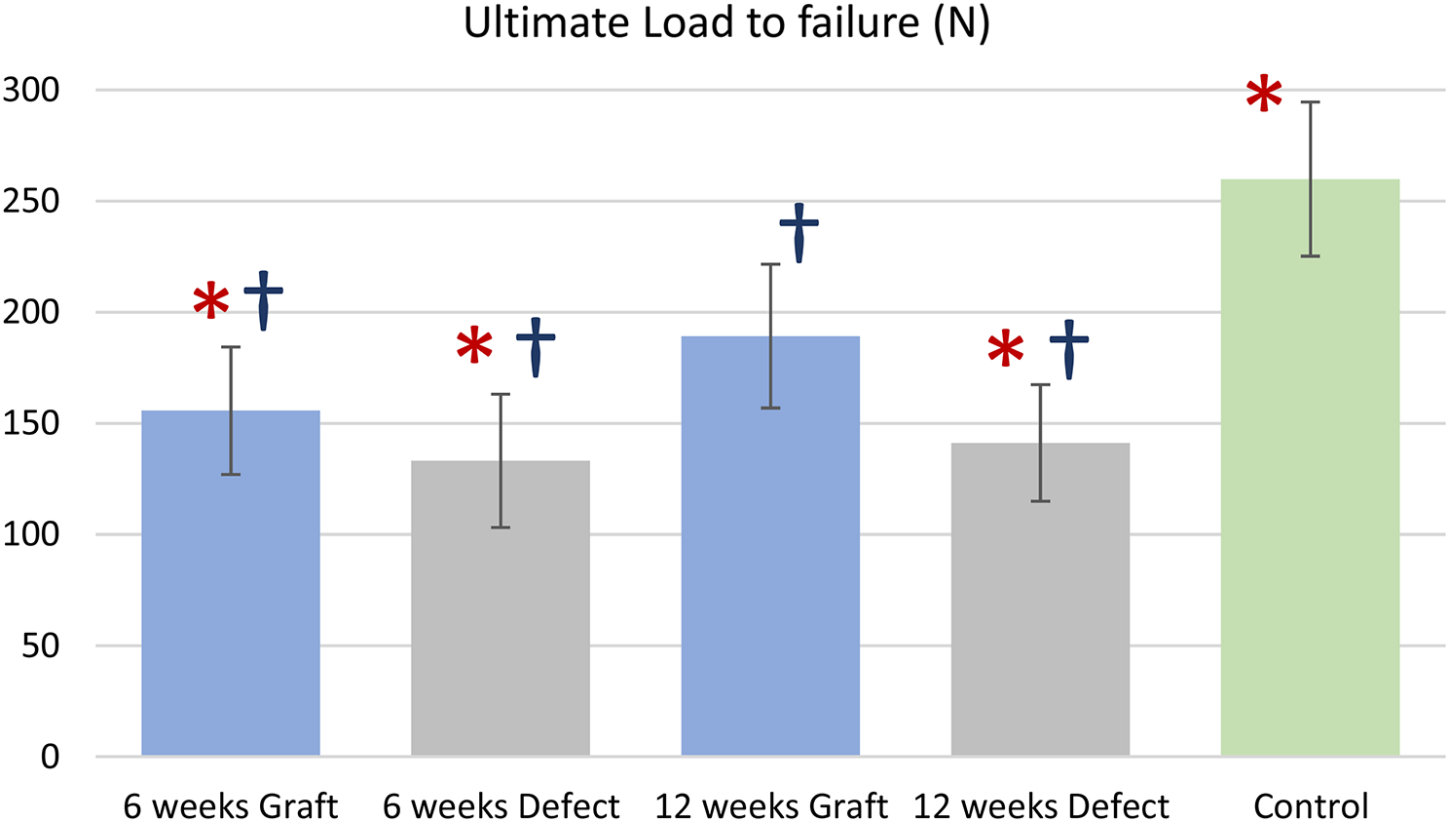
Graph demonstrating mean ultimate load to failure(N) and error bars equal 1 standard deviation of the mean; *p < 0.05 compared with 12-week graft group, †*p* < 0.05 compared with control group

At 6 weeks after reconstruction, the ultimate failure load was measured as 155.8 ± 28.7 N in the graft group and 133.3 ± 30.0 N in the defect group; however, no statistically significant difference was observed. In contrast, at the 12-week mark post reconstruction, the ultimate failure load significantly differed (*p* < 0.05) between the graft group (189.4 ± 32.3 N) and the defect group (141.4 ± 26.2 N). The failure load of the graft group at 12 weeks was also significantly higher than the failure load of the graft group at 6 weeks (*p* < 0.05). In the control group, the failure load was 260.0 ± 34.8 N and significantly higher (*p* < 0.05) than that in all the other groups (Fig. 6).

**Fig. 7:**
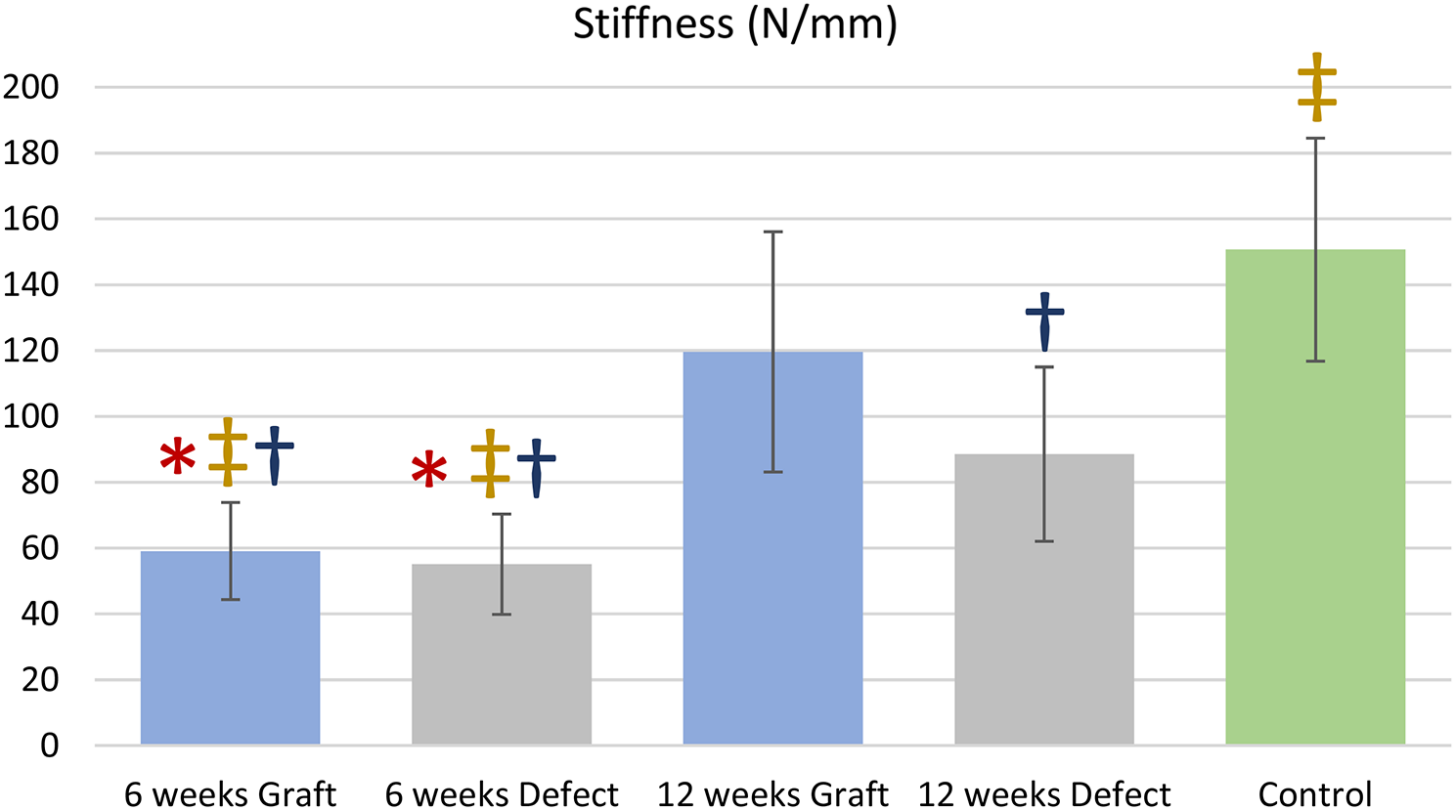
Graph demonstrating mean stiffness(N/mm) and error bars equal 1 standard deviation of the mean; *p < 0.05 compared with 12-week graft group, ‡*p* < 0.05 compared with 12-week defect group, †*p* < 0.05 compared with control group

The linear stiffness of the graft group increased significantly (*p* < 0.05) from 59.1 ± 14.9 N/mm at 6 weeks to 119.6 ± 36.7 N/mm at 12 weeks. Similarly, the same value in the defect group showed a significant increase (*p* < 0.05) from 55.1 ± 15.2 N/mm at 6 weeks to 88.6 ± 26.5 N/mm at 12 weeks. The control group had a linear stiffness of 150.6 ± 33.8 N/mm, which was significantly greater (*p* < 0.05) than that of all groups, except for the graft group at 12 weeks (Fig. 7).

### Site of failure

**Fig. 8:**
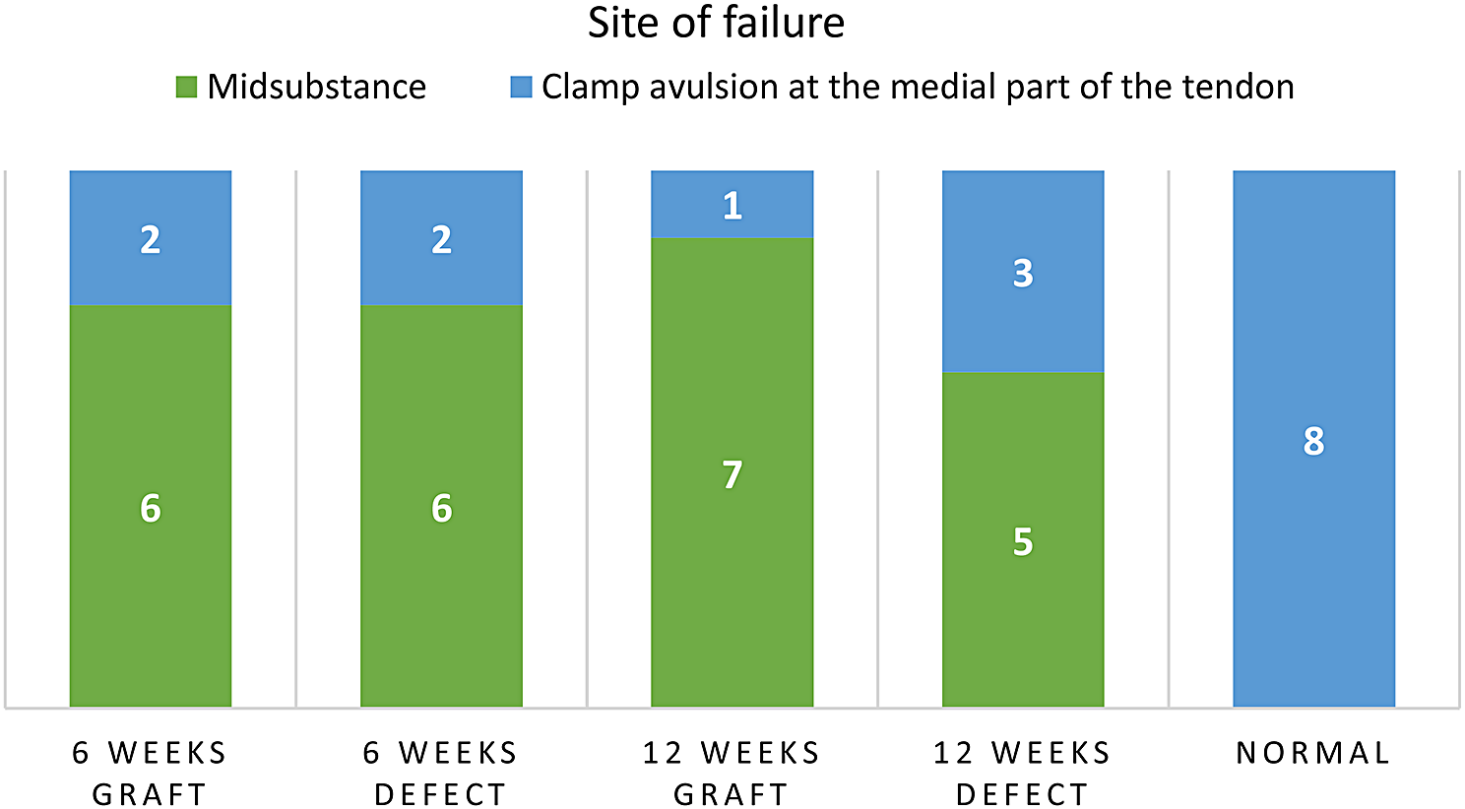
Graph demonstrating site of failure

The site of failure is summarized in Fig. 8. Interestingly, none of the specimens exhibited failure at the site of tendon-bone junction.

There were no statistically significant differences in the site of failure between the graft and defect groups across all time points. Clamp avulsion at the medial part of the tendon (proximal to muscle belly) was associated with significantly higher (*p* < 0.05) linear stiffness.

### CT examination

**Fig. 9:**
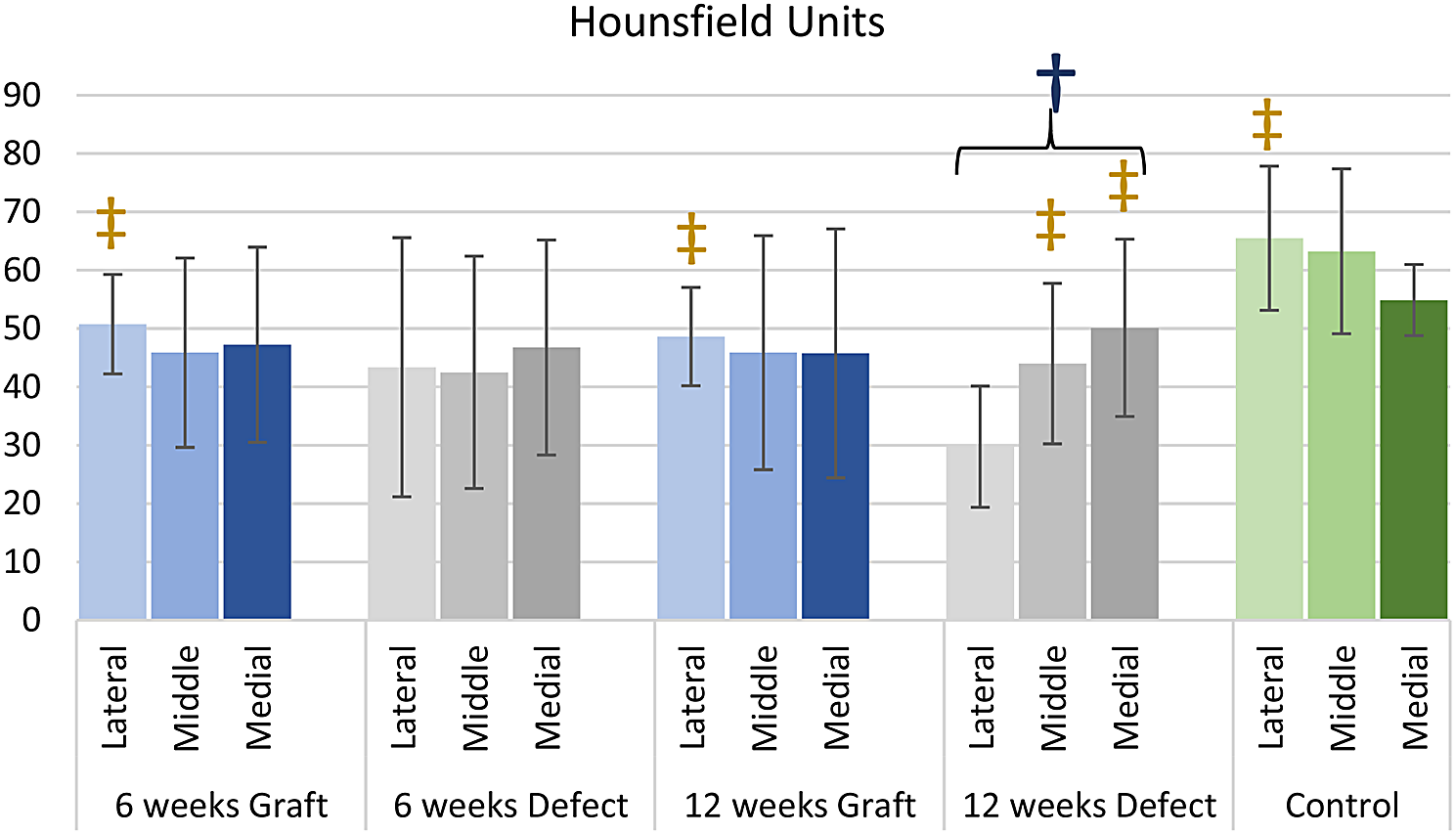
Graph demonstrating mean CT attenuation values, measured in Hounsfield units (HU) and error bars equal 1 standard deviation of the mean; ‡*p* < 0.05 compared with the lateral quarter of 12-week defect group, †*p* < 0.05 compared with the mean intramuscular fat signal of the subscapularis muscle control group

The CT attenuation values in HU, which indirectly indicate intramuscular fat concentration, are demonstrated in Fig. 9.

At 12 weeks the defect group demonstrated significantly lower attenuation (*p* < 0.05) than the control group, reflecting greater fat content. Interestingly, no significant difference was evaluated between the graft groups at 6 and 12 weeks, and the control group, indicating that reconstruction with the graft hindered the progression of fat accumulation.

**Fig. 10:**
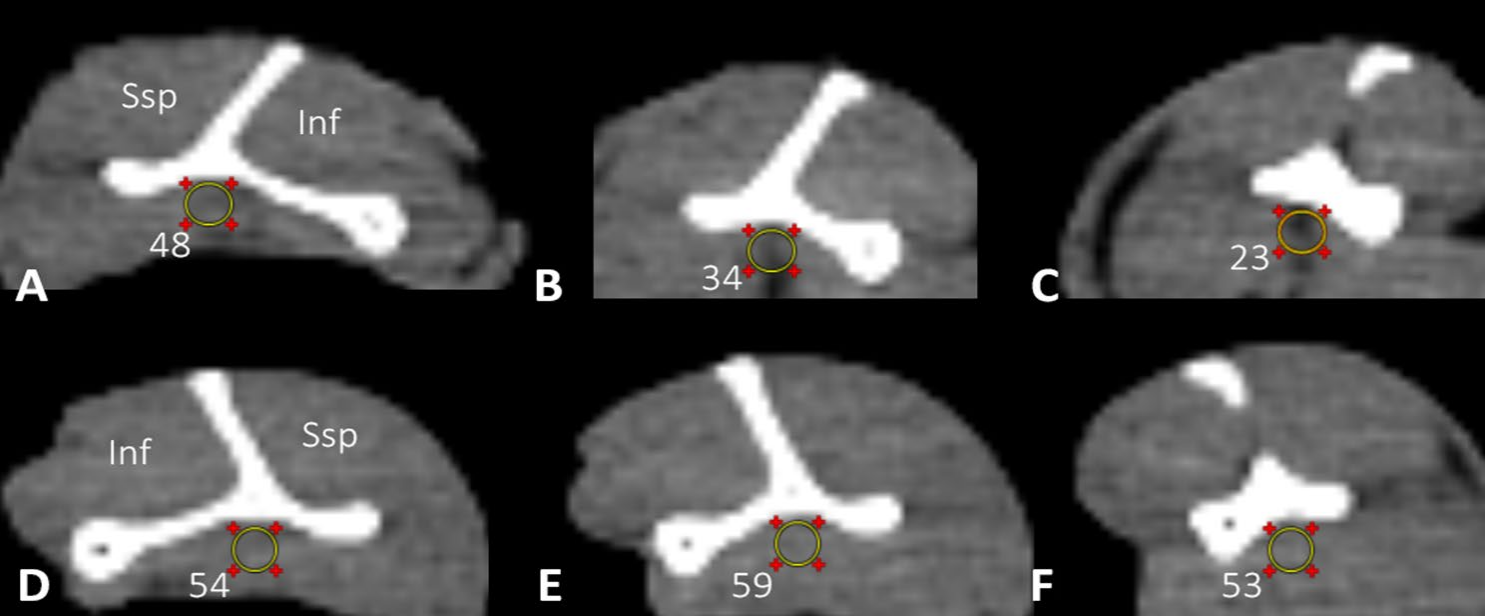
Sagittal CT images from a 12-week defect group specimen (left shoulder) at (**a**) medial quarter, (**b**) middle half and (**c**) lateral quarter. Sagittal CT images from a 12-week graft group specimen (right shoulder) at (**d**) medial quarter, (**e**) middle half and (**f**) lateral quarter. The yellow circle represents the 10-mm² circular region in which the Hounsfield units (HU) were calculated. Lower HU values correlate with greater fat accumulation. The muscle bellies of supraspinatus (Ssp) and infraspinatus (Inf) are also demonstrated

The intramuscular fat accumulation in the lateral quarter was significantly greater (*p* < 0.05) in the 12-week defect group than in the lateral quarter in the 12-week graft group. The CT intramuscular fat signal in the lateral quarter of the 12-week defect group was also significantly greater (*p* < 0.05) than that in the same quarter in the control group (Fig. 10).

CT also depicted a gradient of intramuscular fat accumulation within the 12-week defect group, with a significantly lower attenuation (*p* < 0.05), indicating greater fat content, at the lateral quarter compared with the middle half and medial quarter. No gradient in the attenuation signal was found in any of the other groups or the control group.

Spearman’s rank correlation coefficient showed no relationship between the CT attenuation signal and ultimate load to failure or stiffness.

### Histological examination

#### Control group

**Fig. 11:**
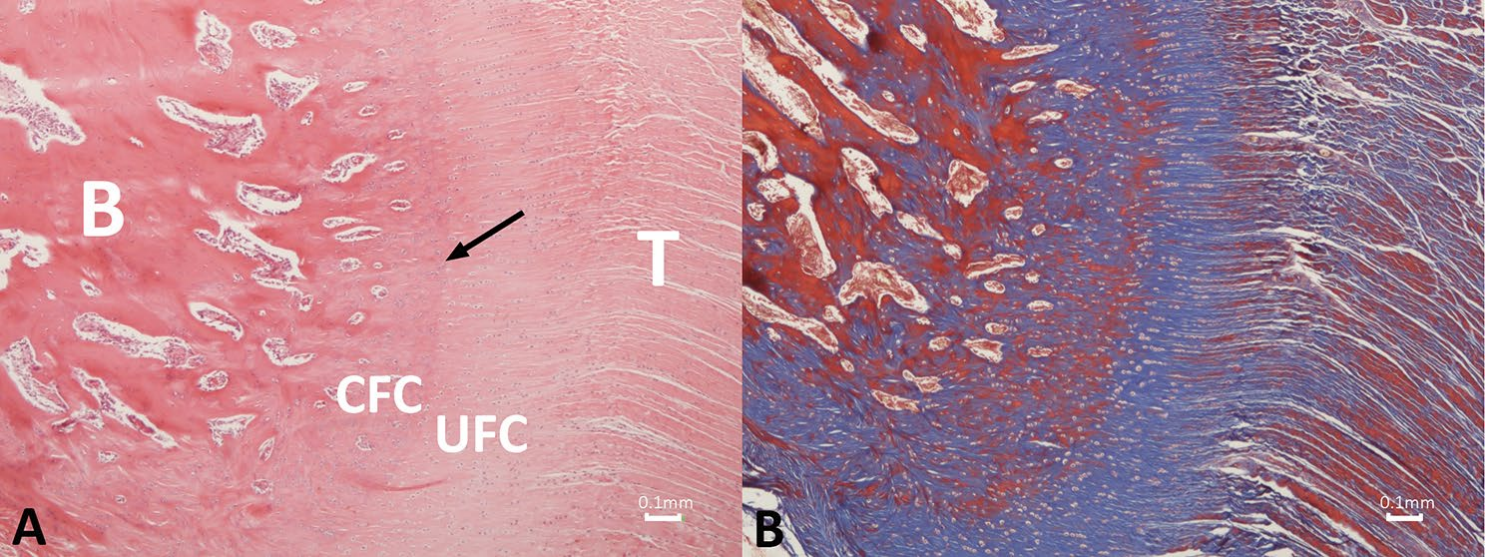
Photomicrograph depicting the histologic structure of the normal rabbit subscapularis bone-tendon junction consisting of four different regions: the tendon proper (T), uncalcified fibrocartilage (UFC), calcified fibrocartilage (CFC) and bone (B) in the control group. The tidemark (arrow), a prominent demarcation, is seen between the uncalcified and calcified fibrocartilage. Fibrochondrocytes, rounded and lying in lacunae within cartilage matrix, are arranged in columns, while the collagen fibers exhibit a parallel orientation. (**a**) Hematoxylin and eosin, original magnification × 40; (**b**) Masson’s trichrome staining, original magnification × 40

The bone‒tendon enthesis displayed a clearly visible normal four-zone structure, including the tendon proper, uncalcified fibrocartilage, calcified fibrocartilage, and bone. The tidemark zone, which separates the two layers of fibrocartilage, and Sharpey’s fibers, which anchor the tendon to the bone, were also evident. Additionally, the cartilage cells were arranged in columns, while the collagen fibers exhibited a parallel arrangement. Furthermore, no inflammatory cells were detected in the area (Fig. 11).

### Graft group

**Fig. 12:**
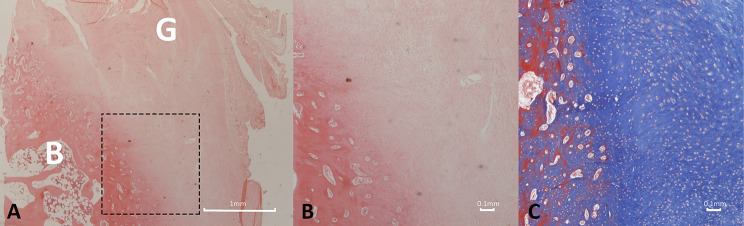
Photomicrograph depicting the histologic structure of the tendon-bone region in the 6-week graft group. A poorly organized pattern of reparative collagen fibers is observed bridging the gap between the graft and bone trough. A significant degree of cellularity, along with mild vascularity and inflammation around the graft is present. The rectangular box indicates the area of higher magnification. G: graft; B: bone. (**a**) Hematoxylin and eosin, original magnification × 20; (**b**) Hematoxylin and eosin staining, original magnification × 40; (**c**) Masson’s trichrome staining, original magnification × 40

In the 6-week graft group, a moderate degree of collagen fibers was observed bridging the gap between the graft and bone trough. These fibers, located at the site where they anchored into the newly formed callus, resembled Sharpey’s fibers. However, only a portion of the newly formed fibers exhibited a parallel orientation. There were few vascular structures present and a mild degree of inflammation around the graft. Within the tendon–bone junction region of the graft group, granulation tissue filled the area, containing a significant number of cells (Fig. 12).

In the 12-week graft group, significant changes were observed in the tendon–bone junction region. A typical columnar arrangement of chondrocytes, along with the presence of the tidemark, was evident. Moreover, the number of collagen fibers increased, and their orientation exhibited greater parallel arrangement, notably resembling normal structures that effectively anchored the tendon to the bone.

**Fig. 13:**
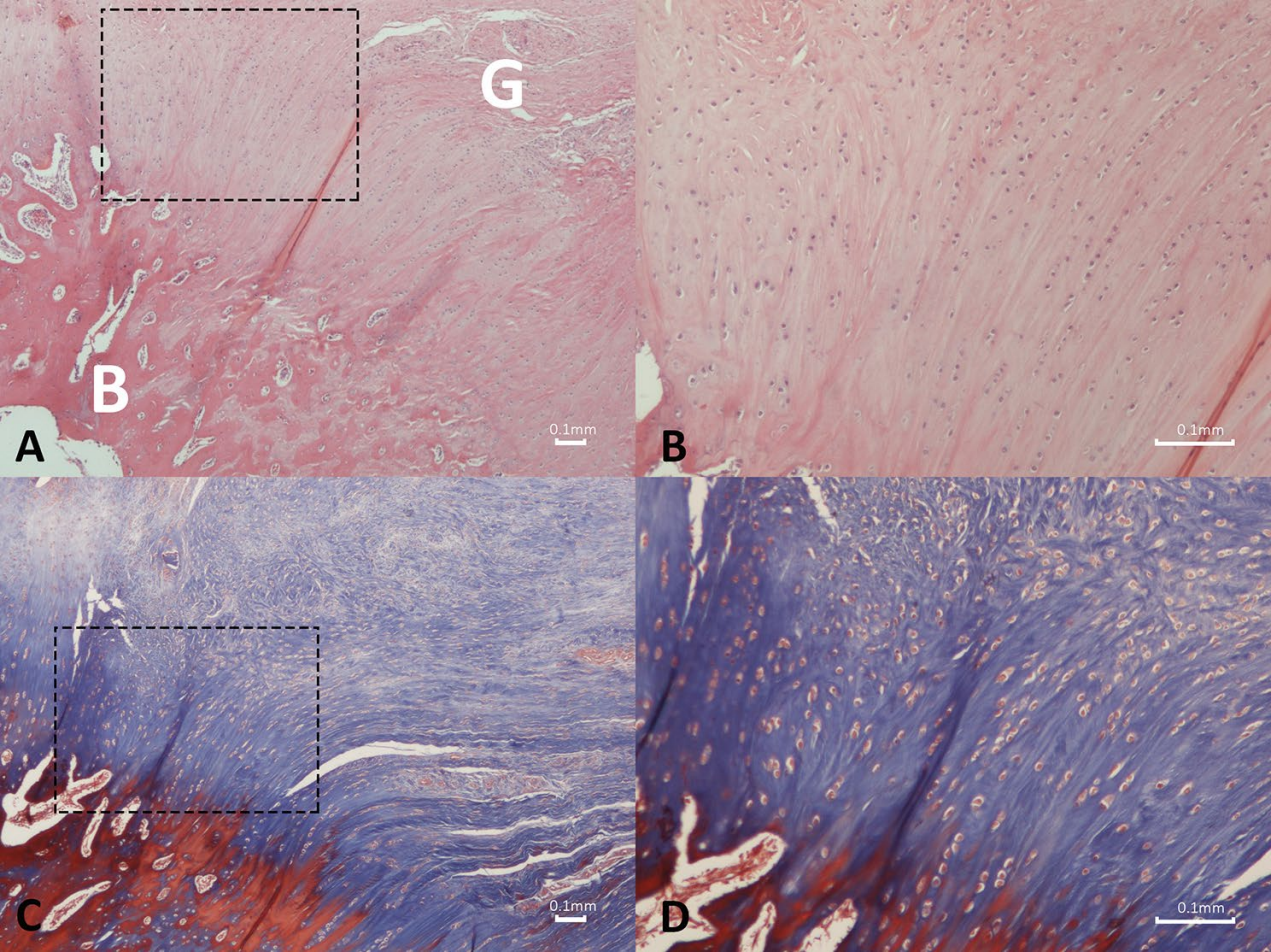
Photomicrograph depicting the histologic structure of the tendon-bone region in the 12-week graft group. Chondrocytes can be seen in rows, while collagen fibers exhibit parallel arrangement. In addition, increased presence of vascularity and diminished inflammatory response is observed. The rectangular box indicates the area of higher magnification. G: graft; B: bone. (**a**) Hematoxylin and eosin, original magnification × 40; (**b**) Hematoxylin and eosin staining, original magnification × 100; (**c**) Masson’s trichrome staining, original magnification × 40; (**d**) Masson’s trichrome staining, original magnification × 100

Furthermore, there were varying degrees of increased cellularity and vascularity (Fig. 13).

### Defect group

**Fig. 14:**
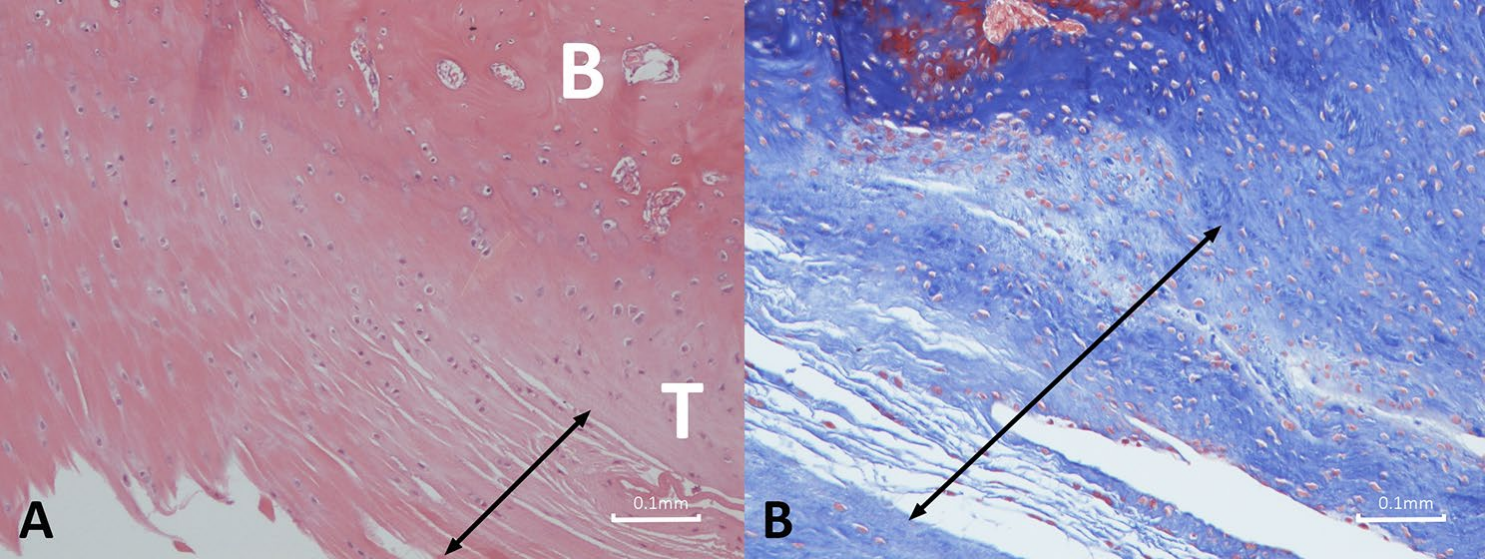
Photomicrograph depicting the histologic structure of the tendon-bone region in the 6-week defect group. Fibrous connective tissue with poorly organized collagen fibers (arrow), increased cellularity and vascularity are evident. T: Tendon; B: bone. (**a**) Hematoxylin and eosin, original magnification × 100; (**b**) Masson’s trichrome staining, original magnification × 100

At 6 weeks, there was tight fibrous connective tissue present in the tendon defect area, and the defect remained visible. This tissue exhibited increased collagen fibers, cellularity, and neovascularization. Moreover, the collagen fibers had poor organization and continuity. Notably, there was no evidence of an inflammatory response (Fig. 14).

**Fig. 15:**
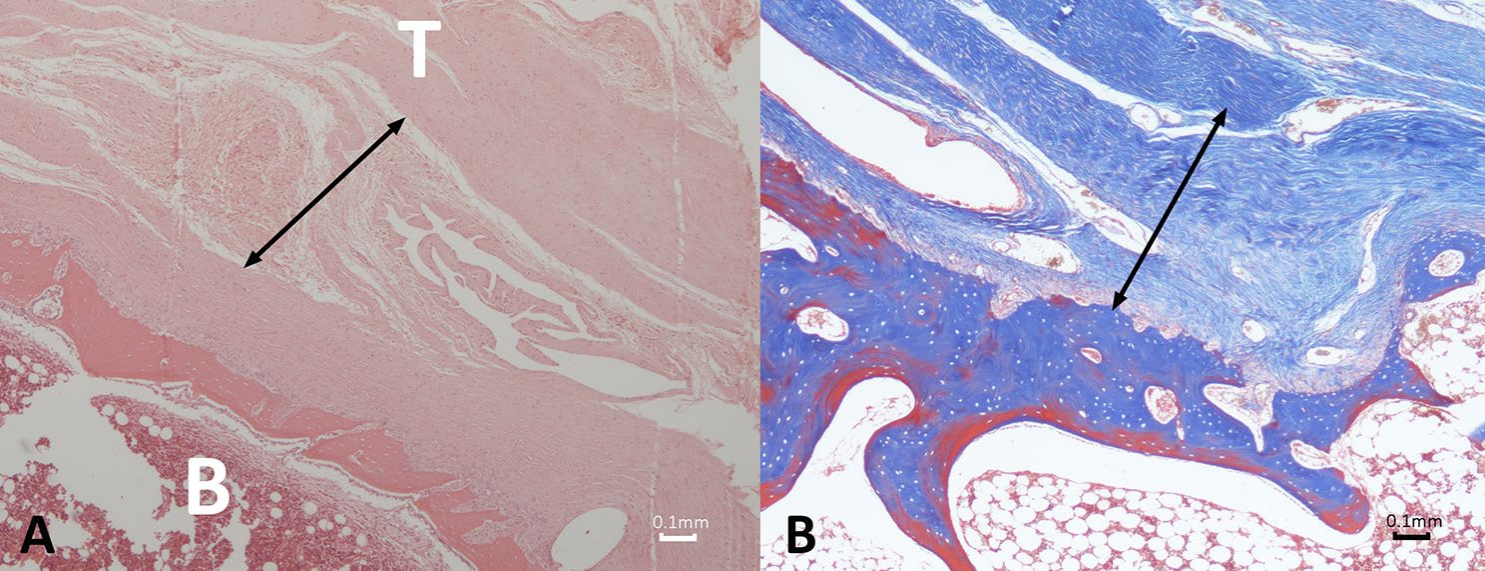
Photomicrograph depicting the histologic structure of the tendon-bone region in the 12-week defect group. Disorganized fibrous connective tissue with no formation of fibrocartilage is shown (arrow). T: Tendon; B: bone. (**a**) Hematoxylin and eosin, original magnification × 40; (**b**) Masson’s trichrome staining, original magnification × 40

At 12 weeks, the fibrous tissue became more prominent, with disorderly alignment of collagen fibers, absence of fiber continuity, and no formation of fibrocartilage (Fig. 15).

## Discussion

Our study confirmed that the intrasynovial flexor tendon autograft effectively enhanced biomechanical properties, hindered fatty infiltration, and promoted healing when bridging a chronic large RC defect in a rabbit SSC model, supporting our initial hypotheses. To our knowledge, no study to date has investigated the use of an intrasynovial flexor tendon autograft for RC tear interposition reconstruction.

Large and massive RC tears, especially those with muscle atrophy and fatty infiltration, are difficult to repair by suture repair alone; thus, multiple grafts have been proposed as augments to enhance biological healing [7]. Ideally, an RC graft should be safe and have similar mechanical and biological properties to the native tendon. A number of degradable synthetic materials, such as polycaprolactone (PCL) scaffolds [22], polylactic acid (PLA) scaffolds [23], and polylactide-co-glycolide (PLGA) porous scaffolds [24], have been tested in RC tears in animal models, and although favorable histological and biomechanical outcomes have been demonstrated, their degradation mechanism and the results of their metabolites to the surrounding tissues are not fully understood. Allogeneic tissue grafts, such as allograft fascia lata [25], have also been studied, demonstrating that they can aid in the regeneration of new tissue while increasing the biomechanical properties of the tendon-graft construct. However, future investigation is needed to remove immunogenicity of the graft while retaining its extracellular matrix structure to avoid the risk of postoperative infection [26]. Collagen and decellularized matrix scaffolds also comprise a vast study area in the field of RC regeneration, especially in rabbits. These biomaterials alone or with the addition of growth factors or cells have demonstrated that they have the ability to enhance collagen fiber formation at the bone‒tendon enthesis while also improving the mechanical properties of the augmented tendons compared to those repaired with simple sutures [7]. Nevertheless, the effect of the host immune response upon residual cross-species antigens on the scaffold, the rate and effects of scaffold degradation, and the mechanism of interaction of growth factors remain largely unexplored [27].

Autologous tendon grafting represents a more traditional approach to RC tendon reconstruction, and a variety of tissues have been studied thus far in animal models: fascia lata [25, 28–30], periosteum [31], bursal tissue [32], tendon-bone composite autografts [33, 34] and free tendons [34]. All studies using the aforementioned autografts have demonstrated that autologous tissue is a patch that acts as an ideal scaffold with excellent biocompatibility, allowing for better tendon enthesis healing and improvement of the biomechanical properties without causing inflammation. To date, all tendon grafts that have been studied in RC tear animal models represent extrasynovial tissue. On the other hand, intrasynovial grafts, before transplantation, are covered with a loose connective tissue lubricating layer, and are surrounded with synovial cells, that may be associated with less adhesion formation [35] when used to treat intrasynovial defects. Therefore, in our study, we chose to investigate the biomechanical, CT, and histological outcomes of the intrasynovial FDP tendon autograft when used as an interposition graft for patching chronic large defects in the SSC rabbit model.

Pan et al. [21] investigated the postoperative outcomes of acute infraspinatus defect repair with a decellularized tendon slice graft as an interposition patch in a rabbit model, and they reported a significant increase in the ultimate tensile load in the graft group between 4 and 12 weeks. Additionally, they found that stiffness in the graft group at 12 weeks was greater than that in the defect group at 12 weeks. Their histologic analysis at 12 weeks revealed promising cell ingrowth and tissue integration between the graft and host tissue. Kataoka et al. [28] described the early healing process of fascia lata autograft augmentation in an acute infraspinatus repair rabbit model and found a significant increase in ultimate failure load at 4 weeks compared to the simple repair group. Similarly, histologically, they showed that the tendon maturity score of the fascia lata group was significantly better than that of simple repair at the same time point. Xu et al. [36] reported on the results of bridging repair of chronic supraspinatus massive tears in rabbits with 3 different autografts: rectangular fascia lata patch, banded fascia lata graft, and banded patellar tendon graft. In their study, they evaluated the effectiveness of the dual-suspensory reconstruction technique using banded grafts and found a significantly greater mean failure load for the banded grafts at 12 weeks compared to the simple patch graft. In line with their biomechanical evaluations, the fascia lata patch displayed poorer graft substance remodeling and healing interface than the banded grafts at the same time point. In our study, 12 weeks after the second procedure, there was a significant superiority in ultimate failure load in the graft group when compared to the unrepaired defect group. In addition, the graft group at 12 weeks demonstrated no significant difference in stiffness when compared to the normal nonoperated tendons and revealed histological findings of gradual integration to the host tissue.

Muscle atrophy and fatty infiltration following chronic RC tears can be measured using various imaging techniques: CT [37], MRI [38] and ultrasonography [39]. Trudel et al. [20] quantified fat accumulation and its temporal progression after 4, 8, and 12 weeks in rabbit supraspinatus muscles after detachment. They observed that significant fat accumulation starts as early as 4 weeks after tendon detachment, both within and around the supraspinatus muscle, and progresses through the first 12 weeks. Additionally, they reported that at all time points after tendon detachment, an increasing medial-to-lateral intramuscular and extramuscular fat accumulation gradient appeared. Most importantly, they found that a strong CT-to-histology correlation exists regarding muscle tissue and fatty infiltration evaluation. Furthermore, the previous authors in a later study [40] evaluated the outcomes of reattaching the supraspinatus tendon at the previously mentioned time points, 12 weeks after the second operation. They reported that histologically, the intramuscular fat had increased in the middle half and lateral quarter of the SSP muscle tissue in the animals that they repaired after 8 and 12 weeks compared to the unoperated control group. In addition, they noted that extramuscular fat increased significantly for all time repair groups when compared to the control group. Similar to their first study, CT attenuation data correlated strongly with the histologic analysis. McClellan et al. [41] evaluated the association between muscle volumetric intensity histograms from microCT images of acute infraspinatus defects in rabbits, with analysis of histologic samples and tensile load to failure tests. They found that a histogram with greater intensity in repaired or contralateral intact shoulders demonstrated a negative correlation with total fat percentage and a positive correlation with ultimate load to failure. In our study, we performed a CT analysis on specimens that we later subjected to biomechanical analysis and thus were not ideal candidates for additional histologic evaluation. Contrary to the findings of Trudel et al., we did not find lower CT attenuation data in the grafted groups compared to the control group at any time point. Wallenberg et al. [42] retrospectively analyzed human shoulder MRIs with partial or full-thickness supraspinatus tears and varying degrees of tendon retraction. They found that the percentage of fatty infiltration increased as the magnitude of tendon retraction increased. Notably, they reported that muscles with less than 10% fatty infiltration exhibited an asymmetric concentration of fat, predominantly in the lateral third of the muscle, while the medial two-thirds of the muscle had a fat content similar to that of healthy controls. Interestingly, we also found in our large subscapularis rabbit model that a chronic tear, if left untreated for 12 weeks, creates fatty infiltration, that is prominent in the lateral quarter of the muscle.

There are several limitations in our study that should be acknowledged. First, this study was conducted in rabbits, which are quadruped animals that use their upper limbs for weightbearing. It is important to note that during the course of the experiment, no immobilization or protection of the repair was performed. Furthermore, the healing process and anatomy of the shoulder differ between humans and rabbits, which limits the direct clinical application of our findings. Second, the number of specimens used for histologic examination was small. As a result, quantitative measurements of the histologic findings with statistical analysis could not be executed, limiting the strength of our conclusions in this regard. Third, a comparison group with other types of grafts was not included in our study due to the primary focus on evaluating the biomechanical properties and healing potential of the intrasynovial flexor tendon autograft. Therefore, future investigations should be considered to evaluate whether the graft we tested exhibits superior biomechanical properties and healing potential compared to alternative graft options. Fourth, after sacrificing the animals, we did not keep the SSC perimuscular tissue, and therefore, we did not evaluate extramuscular fat accumulation. Fifth, we did not histologically calculate intramuscular fat accumulation but rather used CT data to indirectly measure the fat content of SSC muscle, based on the previous studies of Trudel et al. [20, 40]. Finally, our study focused specifically on the treatment of large rotator cuff tears. Further studies examining the efficacy of this treatment in the context of massive rotator cuff tears may be necessary to assess its effectiveness in an even more challenging clinical scenario.

## Conclusion

The intrasynovial flexor tendon autograft in the interposition reconstruction of chronic large RC tears resulted in increased mechanical properties, followed by no progression in fatty infiltration and enhanced tendon-bone healing. This animal study suggests that the intrasynovial flexor tendon autograft holds promise as an effective interposition graft for the reconstruction of chronic large RC tears, as it improves the biomechanical and biological properties of the repaired tendon. However, further investigation is required in preclinical large animals, which may represent a valuable avenue of research in the clinical setting for patients with RC tears.

## Data Availability

The datasets used and/or analyzed during the current study are available from the corresponding author upon reasonable request.

## Abbreviations

RC: rotator cuff
SSC: subscapularis
CT: computed tomography
CSA: cross-sectional area
HU: Hounsfield unit
LVDT: linear variable differential transformers
PCL: polycaprolactone
PLA: polylactic acid
PLGA: polylactide-coglycolide
FDP: flexor digitorum profundus
